# *Anaplasma marginale*: Diversity, Virulence, and Vaccine Landscape through a Genomics Approach

**DOI:** 10.1155/2016/9032085

**Published:** 2016-08-17

**Authors:** Rosa Estela Quiroz-Castañeda, Itzel Amaro-Estrada, Sergio Darío Rodríguez-Camarillo

**Affiliations:** Unidad de Anaplasmosis, Centro Nacional en Investigación Disciplinaria en Parasitología Veterinaria, INIFAP, Carretera Federal Cuernavaca-Cuautla 8534, 62574 Jiutepec, MOR, Mexico

## Abstract

In order to understand the genetic diversity of* A. marginale*, several efforts have been made around the world. This rickettsia affects a significant number of ruminants, causing bovine anaplasmosis, so the interest in its virulence and how it is transmitted have drawn interest not only from a molecular point of view but also, recently, some genomics research have been performed to elucidate genes and proteins with potential as antigens. Unfortunately, so far, we still do not have a recombinant anaplasmosis vaccine. In this review, we present a landscape of the multiple approaches carried out from the genomic perspective to generate valuable information that could be used in a holistic way to finally develop an anaplasmosis vaccine. These approaches include the analysis of the genetic diversity of* A. marginale* and how this affects control measures for the disease. Anaplasmosis vaccine development is also reviewed from the conventional vaccinomics to genome-base vaccinology approach based on proteomics, metabolomics, and transcriptomics analyses reported. The use of these new omics approaches will undoubtedly reveal new targets of interest in the near future, comprising information of potential antigens and the immunogenic effect of* A. marginale* proteins.

## 1. Introduction

Tick-borne diseases are major obstacles and are considered the cause of great economic impact for livestock production [1]. Tick-borne rickettsial diseases are important problems of management in livestock health in Africa, Australia, Asia, and Latin America. Globally, the most important rickettsial disease in cattle is bovine anaplasmosis caused by* Anaplasma marginale*, an infectious, noncontagious disease characterized by progressive hemolytic anemia, abortions, loss of condition, milk production, and death [2, 3]. Clinical disease in most notable in cattle, but other ruminants including water buffalo, bison, African antelopes, and some species of deer can become infected [4].

In endemic areas, indigenous cattle have developed resistance to ticks and anaplasmosis [1]. Control measures currently available include the use of acaricides, animal treatment, chemoprophylaxis, controlled exposure, and vaccination. Most of these approaches only limit losses caused by ticks and tick-borne diseases [5]. The use of acaricides is becoming more problematic due to the selection of tick resistant populations; furthermore the presence of acaricide residues in meat and milk is public health concerns and ultimately can interfere with the enzootic stability making animals susceptible to both anaplasmosis and bovine babesiosis [6]. Chemoprophylaxis (treatment-exposure) and controlled infection (exposure-treatment) may or may not be effective even if carried under strict veterinary supervision. Immunoprophylaxis is then the method of choice for the prevention of infectious diseases [7, 8].

Control of bovine anaplasmosis is, however, compounded by the large antigenic and genetic diversity found in strains from one region to another, within the same herd and even within the same animal [9–12].

Current research efforts aim at new alternatives for designing vaccines including the use of sequencing technologies and omics approaches [13–17].

High-throughput sequencing technologies currently available are fast and inexpensive enough processes and currently included in almost any bacteria related project [18]. Whole-genome sequences (WGS) data provides information of gene repertoire and sequence variation and is also an approach to associate genotype with phenotype [19]. Genomics analyses of these data represent a significant tool to understand the bacterial diversity, their phylogenetic relationships, and the mechanisms related to their vital functions (transmission, pathogenicity, metabolic processes, etc.).

The first complete genome sequence of* Anaplasma marginale* (St. Maries strain) was published eleven years earlier with the promise of better immunogens based on more complete knowledge of the genetic makeup of the rickettsia [20]. In addition to* A. marginale *St Maries genome, the genomes of other rickettsial agents of human and animal importance have been reported and analyzed, including* A. marginale *American strain Florida, Gypsy Plains, and Dawn strains from Australia,* A. marginale *subsp.* centrale* (strain Israel),* A. phagocytophilum*,* Ehrlichia chaffeensis*,* Rickettsia prowazekii*, and* R. typhi* [20–26]. In spite of this wealth of genetic information, development of vaccines that can induce protection against an array of strains of* A. marginale *is still pending.

In this review, we analyze the available information with regard to molecular diversity and variability that make* A. marginale* a rickettsia for which vaccine design has turned very difficult. We also present an update of the information of strains reported worldwide and highlight the relevant information for regional vaccine development. In addition, we focus on the* A. marginale* vaccine approaches through conventional, reverse vaccinology, and omics approaches carried so far. Finally, the immunological effect of* A. marginale* proteins with potential as immunogens is also reviewed.

## 2. Diversity of* Anaplasma marginale*

Characterization of strains from diverse geographical origin of* A. marginale* includes morphology, protein sequence, antigenicity, and their ability to be transmitted by ticks [4, 27, 28]. The genetic diversity of* A. marginale* has been classified by using major surface proteins (MSP) such as MSP1a, MSP4, and MSP5, which are encoded by single genes. These genes have been widely used for molecular characterization of* A. marginale* [4].* msp1a* gene which shows wide genetic diversity has been used for identification of* A. marginale* strains worldwide and is considered a stable genetic marker conserved during acute and persistent rickettsemia in cattle and during multiplication in ticks [27, 29, 30].* msp4* gene, which shows a very low variation index, has also been used as a stable marker for phylogeographic studies [9]. On the contrary,* msp5* is extremely conserved between isolates of* A. marginale* and is not phylogenetically informative but rather used in molecular diagnosis of infection by this rickettsia.* msp5* product MSP5 is highly immunogenic and has also been used for serologic diagnostic of the disease [31].

Several geographic strains of* A. marginale* differing in their biology, genetic characteristics, and transmissibility by ticks have been identified using MSP1a; this protein is composed by a C terminal conserved domain and a N-terminal variable domain composed of one or more peptides of 23 to 31 amino acids very similar among them known as repeats [14–16]. MSP1a has evolved under positive selective pressure of the host immune system and molecular weight difference of the peptides in geographic strains is the result of variations in the numbers of tandem repeat units. MSP1a, used as molecular marker, has provided phylogenetic and evolutionary information about* A. marginale* strains [10].

The genetic diversity of* A. marginale* based on MSP1a has been reported in several countries all over the world [30, 32–37]. A global analysis using 131 strains of* A. marginale* from North and South America, Europe, Africa, Asia, and Australia provided information about the genetic heterogeneity of the rickettsia [30]. de la Fuente et al. using* msp1a* [30] found 79 different repeat sequences in 131 strains, thus corroborating the known genetic heterogeneity of* A. marginale* [38]. Although MSP1a repeat sequences did not group in clusters geographically related or offered phylogenetic relationships, they did provide phylogeographic information, as 78% of the repeat sequences were present in strains from a single geographic region. Some MSP1a repeats clustered and were unique to certain regions such as Italy, Spain, China, Argentina, and South America. Australia was a special case, where a single genotype is found, which suggests that multiple introductions of* A. marginale* strains from different geographic locations occurred in the rest of the continental countries.

These authors also found that repeats 27 and 13 were present in strains from geographic regions as distant as Latin America and South Africa, but with the common tick vector,* Rhipicephalus microplus.* In this case, it is impossible to rule the role of other tick species out or mechanical transmission in the evolution of the rickettsia [30].

A molecular analysis using MSP1a revealed the genetic diversity of Mexican strains of* A. marginale* from different geographic origins. Jimenez-Ocampo et al. [32] reported the presence of repeats, such as F, M, and M in a strain from Ticul, Yucatán, also commonly found in Argentina (F, M, M, and M), Israel (F, M), and Italy (M). Some other Mexican strains including Tizimin, Playa Vicente, and Tlapacoyan share a significant sequence of tandem repeats with Florida strain (A, B, B, B, B, B, and B). Strains from central states of Mexico (Yautepec, Morelos) and states near the Gulf of Mexico (Veracruz, Veracruz) and the West coast (Tepic, Nayarit) have some variants of the repeats *α*, *β*, *β*, and Γ, also found in strains from Argentina and Brazil.

Recently, Castañeda-Ortiz et al. [12] reported 14 new* msp1a* genotypes detected in infected animals from two cattle herds in Mexico, called EV1–12 and LJ1-LJ2.

The wide genetic diversity observed in Mexican strains of* A. marginale* reveals the significant role of cattle movement and reinforces the proposal of regional vaccines to control anaplasmosis.

Phylogeographic analysis of MSP1a has also revealed an association between the first (R1) and last (RL) MSP1a repeat sequences and world ecological regions (ecoregions) specific signatures, which implies a different evolutionary pressure and the MSP1a sequences [39]. The authors found 39 and 28 unique R1 and RL sequences, respectively, of 111* A. marginale* strains. The MSP1a R1 is associated with four ecoregions, each of them with unique repeats sequences (i.e., ecoregion 1: 4, 8, 16, 56, 60, 64, 67, *γ*, *π*, *τ*; ecoregion 2: 28, 48, 53, E, F, Σ; ecoregion 3: 1, 3, 5, 6, 27, 33, 34, 39, M, O, Q, U; and ecoregion 4: I, J, K). The RL MSP1a repeat is also associated with four ecoregions (i.e., ecoregion 1: 8, 9, 12, 15, 59, 61, 66; ecoregion 2: 10, 31, 52, *π*, *β*; ecoregion 3: 3, 7, 35, 37, 38, 44, E, N, P, Q, U, *ρ*; and ecoregion 4: none). This was the first evidence that the evolution of* A. marginale* was linked to ecological traits affecting tick vector performance and how these traits have driven the evolution of vector-borne pathogens.

Recently, Machado et al. [35] reported outbreaks of anaplasmosis in two municipalities of Brazil, Lins and Mambaí. According to the analysis of the tandem repeat structures of MSP1a, nine different strains were found in Lins (*τ*, 10, 15; *α*, *β*, *β*; *α*, *β*, *β*, *β*, 13; *α*, *β*, *β*, 192; *τ*, *β*, 100; *α*, *β*, *β*, Γ; 193, *β*, 100; 191, 13, Γ; and 191, 13, 18) and two in Mambaí (*α*, *β*, *β*, Γ and E, F, *φ*, *φ*, F, F). The limited genetic diversity of* A. marginale* observed in the Mambaí region is attributed to an intensive tick control program prior to the anaplasmosis outbreak among the cattle sampled in this location, and authors believe that transmission occurred mainly through bloodsucking flies instead of tick vectors. The authors also described three new repeats of MSP1a (191, 192, and 193) and associated the *τ*-10-15 and *α*-*β* ^3^-Γ strains with the occurrence of clinical anaplasmosis and mortality in calves, heifers, and lactating cows. As reported before, out of the different strains of* A. marginale* identified worldwide some have been associated with the occurrence of anaplasmosis outbreaks; specifically, the *α*, *β*, Γ and *τ*, 10, 15 strains have been previously described in outbreaks of bovine anaplasmosis in Mexico and Argentina [40, 41].

Likewise, but in Rio de Janeiro in Brazil, Baêta et al. [36] reported two strains of* A. marginale*, AmRio1 and AmRio2, that were isolated and propagated in IDE8 cells from blood of two cattle. One of the isolates, AmRio1, has a new amino acid sequence of the MSP1a tandem repeat (named 162). The authors also performed a phylogenetic analysis using Argentinian and Brazilian strains. They observed that the population of* A. marginale* in both countries form two big clusters: *α* and *τ*; cluster *τ* has wider genetic variability than cluster *α*, suggesting that lineages belonging to these two clusters may be under different sources of selective pressure, specifically host immunity and tick transmission. Then, new combinations of tandem repeats may give these strains adaptive advantages over those pressure factors.

An interesting case was reported by Mutshembele et al. [37]; these authors carried a prevalence analysis and evaluated the diversity and evolution of MSP1a in South African strains of* A. marginale*. They found that tandem repeats 3, 4, 13, 34, Q, and 37 had a high frequency and have been reported in strains of Israel (3, 4), South America (4, 13), and Europe (Q). Repeats 34 and 37 were abundant only in South Africa with rare exceptions. Through a reconstruction of ancestral amino acid sequence, they found that tandem 4 is the ancestral state of all new repeats reported in South Africa. It should be interesting to test whether the tandem repeat 4 from MSP1a reported in Mexican* A. marginale* strains evolved from the South African repeat [41]. The authors suggest that the repeated sequences identified in South Africa may constitute a group of recently evolved tandem repeats, which have not been reported elsewhere [27].

In 2014, Ybañez et al. [42] using MSP1a and GroEL as molecular markers reported high genetic diversity of* A. marginale* in Philippine cattle; this was the first report of* A. marginale* genotypes in Southeast Asia. They identified 20 novel and unique tandem repeat sequences arranged in 44 new genotypes; repeats showed an identity of 90–96% to those found in Mexico, Brazil, Argentina, South Africa, Venezuela, Japan, Israel, China, United States, and Italy. In some samples, multiple infections of even three and four different genotypes of* A. marginale* were observed, being dual infections, the most common cases. The superinfection may be the consequence of a common exposure or source of the infection despite geographical boundaries, the cattle trade, or movement among different islands in the country.

Different structure of tandem repeats of MSP1a reported worldwide is shown in Table 1.

Information available on genetic diversity of* A. marginale* highlights the fact that variation observed is only the result of natural adaptation processes and pressure exerted on the rickettsia* A. marginale* and cattle movements that occurs in a global trade system. So far, the isolation and identification of strains of* A. marginale* provide the information necessary in a wide landscape of how this rickettsia is distributed all over the world to have better control and prevention strategies.

In these latter strategies, vaccination is proposed as one of the most effective tools for the prevention of infectious diseases. Along with the continuous sequencing of genomes, the availability of the information has led to a new paradigm in vaccine development using technologies such as functional and structural genomics [44].

**Figure 1:**
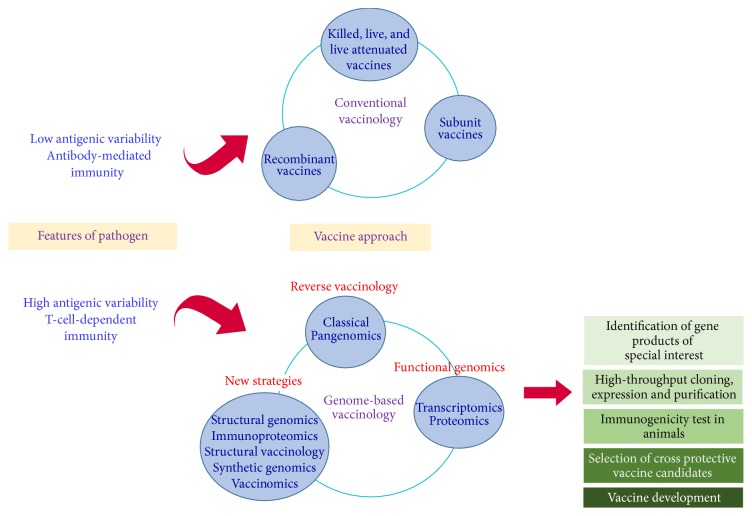
General scheme of* A. marginale* vaccine development using conventional and genome-based vaccinology.

Figure 1 shows a schematic overview of conventional vaccinology versus vaccinology in the genomic era. We present two proposals to follow based on pathogen characteristics in order to achieve potential vaccines and compare conventional vaccinology and genome-based vaccinology approaches as tools to vaccine development. We believe that new strategies, especially those focusing on omics techniques, will lead to a better design of vaccines.

## 3. Vaccine Approach: Conventional Vaccinology of* Anaplasma marginale*

Although* A. marginale* has a global impact on animal health, so far there is no worldwide-accepted vaccine for bovine anaplasmosis.

The first attempt at vaccine was in the early 1900s, with the isolation of* A. marginale *subsp.* centrale*, a less virulent strain that induces cross protection to virulent strains [2].* A. centrale* has been used as a live blood vaccine, for over 100 years, and is currently used in Australia and several African, South American, and Middle Eastern countries including Israel [45]. Although* A. centrale* has been used as live vaccine and gives protection against some strains of* A. marginale*, in countries like Zimbabwe, Paraguay, and Argentina some studies have shown that* A. centrale* provides little to no protection, which means that this rickettsia does not provides 100% protection, maybe probably to dissimilar endemic strains by country and variation in the challenge dose among studies [46–49].

Despite the benefits of this live vaccine, its use represents the risk of cotransmission of other ruminant pathogens (blood-borne pathogens: bovine leukemia virus, unknown or recently emergent pathogens) and hemolytic diseases in calves born to immunized dams, the potential risk of disease induced by the vaccine strains themselves, besides the fact that live vaccines are not licensed for use in many countries, including the United States, Mexico, and European Union [25, 45, 50, 51].

Inactivated vaccines based on the use of the bacteria extracted from bovine erythrocytes are very effective but have disadvantages such as the possible contamination with the erythrocyte membrane antigens and wide antigenic variation between geographic strains [52]; and while they diminish the intensity of clinical signs, they do not prevent infection; thus, animals may remain carriers for the rest of their lives [53].

Rodríguez Camarillo et al. [54] assessed the effect of Yucatan strain, a low virulence* A. marginale* strain, and inoculated 113 susceptible cattle at increasing doses (10^4^–10^10^ infected erythrocytes) of Yucatan strain. Only one animal out of 113 (0.9%) required treatment for clinical disease. These results are comparable with those obtained from other attenuated strain vaccines trials. According to de la Fuente et al. [55], live vaccines result in persistent, life-long infections and allow the maintenance of solid and long-lasting immunity against homologous and heterologous strains. Another naturally avirulent strain of* A. marginale* was reported in Australia by Bock et al. [56], the strain of Dawn. They found that cattle vaccinated with* A. marginale* Dawn strain were strongly immune to challenge with heterologous* A. marginale* isolates. Dawn's virulence was not significantly different between steers vaccinated with Dawn* A. marginale* and those receiving* A. centrale*; in fact, Dawn strain offered better protection than* A. centrale* against virulent heterologous challenge in Australia. These results indicate that this strain could be a useful vaccine alternative in Australia although validation of its safety and protection against African and New World isolates in a large scale should be performed [57].

However, not all the low virulent strains exert a positive effect on cattle. The low pathogenic Brazilian strain UFMG1 has been shown to protect cattle against a high pathogenic Brazilian isolate (UFMG2) [58]; however, this protection was not observed when the geographically distant Israeli* A. marginale* Gonen strain was used to challenge cattle inoculated with UFMG1 [59]. With these results, it is clear that UFMG1 had a negligible effect on disease prevention caused by the geographically distant heterologous Gonen strain and this response may be constrained by limited antibody responses.

The use of killed vaccines is another alternative in conventional vaccinology; these vaccines have advantages over live vaccines; that is, the risk of contamination with undesirable infections is low and the cost of storage is inexpensive; besides, only minimal postinoculation reactions are caused. Some disadvantages are the constant use of boosters, the cost of purification of* A. marginale* from erythrocytes, and the lack of cross protection among isolates from geographically distant areas [60].

## 4. Genome-Based Vaccinology

The completion of the genome sequence of* Haemophilus influenza*, in 1995 (the first bacterial genome sequenced), along with the advances in bioinformatics and sequencing technology set the start of a dramatic boom in the sequencing field [61]. By April of 2016, 8,032 completed sequencing projects (completed and published) and 33,496 permanent drafts were reported [62]; this trend highlights the valuable information contained in the microorganisms genomes and the subsequent possibilities to explore. Currently, genomics-based vaccines projects will increase our knowledge and understanding of microbial physiology, epidemiology, pathogenesis, and protein function and further impact the vaccine design and therapeutic development [63].

Today, the research in omics sciences is moving from a hypothesis-driven to a data-driven approach. The availability of omics data is the result of the acquisition of molecular biology results and represents an unprecedented opportunity and also a major challenge [64].

Once the genome of a pathogen is available, several approaches can be taken to identify vaccine/therapeutic targets: reverse vaccinology, pangenomics, comparative genomics, transcriptomics, functional genomics, proteomics, immunomics, structural genomics, and so forth.

Once the* A. marginale* genome was reported by Brayton et al. [20], the genome-wide screening was the next step. They found two families containing immunodominant proteins: MSP1 and MSP2 superfamilies, both members of the Outer Membrane Proteins (OMPs). These two families comprise more than half of the molecules predicted to be on the surface of* A. marginale* and it was hypothesized that they would be good candidates to induce protection. Indeed, protection was achieved in immunization experiments using native MSP1; however, this protection was not observed using recombinant proteins. Unfortunately, recombinant vaccines failed either due to lack of all MSP1b variants used in the recombinant vaccine construct or their the inability to covalently dimerize as native MSP1 molecules do [65, 66].

Santos et al. [67] provided a successful demonstration of epitope-based vaccines using a functional motif of MSP1 and STSSxL (specifically, Am1, STSSQL and Am2, SEASTSSQLGA) which induced a balanced humoral and cellular immune response in mice. They found that this immunogen significantly induced higher IgG2 than IgG1 response, followed by an increased expression of proinflammatory cytokines such as IL-10, IL-12, IL-8, and TNF-*α* involved in the early response and cytokines involved in the postchallenge stage such as IFN-*γ* and TGF-*β*. The authors demonstrated that immunization with Am1 peptide induced higher expression levels of IL12, IL8, and TNF-*α*, molecules involved in differentiation and maintenance of naïve CD4^+^ T-cells to Th1 cytokines and activation of NK cells to produce IFN-*γ* and other Th1 cytokines related to innate and adaptive immunity. In contrast, the response postchallenge of Am2 peptide revealed an upregulation of IL-10 and a weak upregulation of IFN-*γ*. This work is an example of how epitope-base vaccines could be a viable alternative to induce protective immunity against bovine anaplasmosis.

Control of bovine anaplasmosis is made difficult by the genetic variability mechanisms* A. marginale* uses to evade the immune system of the host [68]. An example of this is the appearance of MSP2 and MSP3 variants, which create a wide repertoire of expression site variants through segmental gene conversion [69, 70]. Nevertheless, immunization with native purified MSP2 containing a wide number of variants did not confer protection to cattle challenged with* A. marginale* expressing the same variants as in the immunogen [71].

Additionally, MSP2 and MSP3 are variable among strains with strain-specific alleles encoding structurally and antigenically distinct proteins; thus these proteins are poor candidates for vaccine development [72].

While bacterial pathogens express surface exposed protein complexes structurally and functionally involved in the infective process and many are well characterized, the search for vaccine candidates in* A. marginale* that influence the host immune system still remains [73].

The lack of success using dominant antigens has led vaccine development to focus on subdominant outer membrane proteins (OMP) antigens; however, the challenge still is to select the best candidates for testing in immunization and challenge experiments in order to develop an effective vaccine [13].

Ducken et al. [13] cloned and sequenced genes encoding major subdominant components of the outer membrane from geographically diverse strains. They reported that AM202, Am936, AM854, and AM1096 were recognized by IgG from animals immunized with outer membranes and shown to be protected from challenge; the highest antibody titers and consistent recognition among vaccinates were directed to AM854 and Am936. The animals immunized with recombinant AM854 and AM936 and challenged had similar IgG and IgG2 responses to both proteins. As such, the possible utility of these two proteins as effective vaccine antigens cannot be dismissed.

This approach represents a progress in the search of membrane protein formulations that may have an important role in a protective immune response in immunized animals. Yet, not all proteins are equally capable of inducing protective immunity. AM779 is a highly conserved but minor component of* A. marginale* and it is a subdominant protein of* A. marginale* located in the outer membrane that is not associated with protective immunity [74].

While It seemed that protein complexes or the outer membrane extracts could be used as vaccine candidates given to the protection observed, it is difficult and expensive to isolate them, making it impractical for development and implementation in vaccination programs. In contrast, individual proteins, easily cloned and expressed and adapted for use in subunit vaccines have not shown a significant protection [75]. The effect of the protein complexed as immunogens strongly suggests that new vaccines candidates may work better as a complex instead of as free proteins.

Once the surface expressed proteins were characterized, these complexes were used as an immunogen to test the protective immunity induced by whole outer membranes. Noh et al. [76] induced protection against high-level bacteremia and anemia upon* A. marginale* challenge of cattle and effectively summed up the protection induced by immunization with whole outer membranes.

Noh et al. [75] tested outer membrane-based immunogens to determine whether membrane context affected immunogenicity and the capacity to induce protection. The first immunogen was composed of a complex of outer membrane proteins linked by covalent bonds and known to be protective. The second immunogen was derived directly from the first one, but the proteins were individualized rather than linked. The authors stated that two common features of these effective immunogens were the presence of multiple antigens and the maintenance of spatial relationships among the antigens comprising the immunogen [75]. They also found that the antibody response induced by the linked immunogen was of greater magnitude than that induced by the unlinked-proteins immunogen. These authors believe that the differences in protein content between immunogens may play a significant role in recognition process by B cells activated by helper T-cells. Despite this difference in magnitude, both immunogens induced protective immunity in animals indicating that protective epitopes were present in both antigens and significantly protected animals from challenge.

These findings suggest that future studies to identify protective antigens can consider testing of individual or groups of candidate proteins and arrange only those protective antigens into high molecular weight complexes as a tool to enhance their immunogenicity [75].

## 5. Immunogenic Effects of* A. marginale* Proteins

Derived from the sequence of the* A. marginale* genome, a number of putative surface proteins have been predicted through bioinformatic analysis. These proteins are proposed as potential targets of the immunoprotective response in cattle for the development of a recombinant vaccine. Based on empirical evidence that has shown immunity in cattle, induced by exposure to crosslinked outer membrane fragments and bioinformatics analysis of proteins potentially expressed in the outer membrane with a probable functional role, of which there is no previous information available, Ducken et al. [13] chose six proteins, AM202, AM368, AM854, AM936, AM1041, and AM109, to be compared between distinct geographical strains. Those most highly conserved were recognized in their recombinant form by IgG from animals immunized with outer membranes. In spite of higher recognition titers and IgG and IgG2 production, animals immunized with AM854 or AM936 developed higher bacteremia and anemia after challenge than the adjuvant-only controls [13]. This observation reinforces the notion that surface exposure alone is not sufficient to predict the protective function of a protein and additional elements must be considered.

Protective responses against several bacterial pathogens including* A. marginale* are based on CD4^+^ T-cell action. Cattle immunized with whole initial bodies from a Mexican strain of* A. marginale* were challenged and shown to be tolerant to the infection. In order to identify the molecules involved in protection response, protein recognized by IgG2 produced after immunization protocol were isolated, and MSP1, MSP2, MSP5, and other putative MPs were identified [14]. Proteins not previously reported were recuperated, although whole initial bodies induced immunity in this study, and additional information should be obtained in order to determine each protein role in immunity induction.

In other experiments, a fraction enriched with* A. marginale* outer membranes was found to induce complete protection against homologous experimental infection, elicit CD4^+^ T-lymphocyte proliferation, and IgG2 production [76, 77]. Analysis by two-dimensional electrophoresis, mass spectrometry, and genomic mapping of this outer membrane immunogen identified more than 20 proteins. Native proteins VirB9, Virb10, and CTP reacted with immune bovine sera. An in depth bioinformatic analysis on subdominant antigens determined that these antigens were also outer membrane surface proteins [78].

Analyses based on highly conserved sequences, potential functional role, and surface localization led to the use of type IV secretion system proteins and conjugal transfer protein (CTP) as candidates components in vaccine design. The use of native protein preparations, however, is an obstacle in the production of nonviable commercial vaccines. Therefore, recombinant VirB9, VirB10, and CTP were produced in* E. coli*, and all proteins were able to stimulate T-lymphocyte proliferation and gamma interferon secretion [16]. Furthermore, these recombinant proteins reacted with IgG2 from outer membrane-immunized cattle. It has been well established that IgG2 subclass is associated with Th1 protective immunity in bovine anaplasmosis [14, 77].

On the other hand, adaptive immune responses involving specific MHC molecules and their interaction with T-cell receptors are an early step for processing and elimination of pathogens. Two MHC class II proteins are expressed in cattle, DR, and DQ, and antigenic peptides are classically presented by monomorphic DRA with polymorphic DRB molecules to T-cells [79, 80]. Therefore, the characterization of relevant alleles during* A. marginale* antigen presentation is significant for response modulation and vaccine design. In this way, VirB9-1, VirB9-2, and VirB10 overlapping peptides were assayed on peripheral blood mononuclear cells from varying and heterozygous MHC class II bovines [81], and the authors reported several combinations of MHC alleles recognized by the peptides, mainly alleles prevalent in Holstein cattle. However, these results are most likely influenced by the population under study. Despite the above-reported results, additional assays need to be carried before practical use of VirB9 and VirB10 proteins can be achieved.

Type IV secretion system members such as VirB11 and VirD4 are not predicted as surface proteins; nevertheless they could play a relevant function and may be part of a heterogeneous vaccine [16].

While many investigations have focused on major surface proteins as immunogenic molecules, other predicted proteins have also shown potential at inducing specific immune responses.* A. marginale* characteristic wide diversity, high variability, immune evasion mechanisms, and unknown interactions with the host, however, have made it very difficult to design efficient immunoprophylactics to prevent the disease.

Furthermore, in order to avoid immune response variations in testing potential immunogens, experimental designs should include larger numbers of animals. These type of experiments are very costly and the criteria for selecting potential antigen should be carefully revised.

The identification of successful targets of the protective immune response against* A. marginale* is a great challenge, functional elements remain unknown despite the publication of* A. marginale* genome sequences, and further efforts are required in order to complement bioinformatics analysis if an effective vaccine is to be achieved. As efforts to design a vaccine that will induce solid and long-lasting immunity against a wide array of diverse* A. marginale,* new approaches have been tested, including synthetic and truncated peptides, overlapping peptides, and more recently, while not in the field of anaplasmosis, the use of multiple antigenic peptides (MAPs). MAPs are peptides that are branched artificially, in which Lys residues are used as the scaffolding core to support the formation ≤8 branches with varying or the same peptide sequences [82]. MAPs, if used as immunogens, present many advantages over conventional vaccines; they can include one or more relevant epitopes of a single or multiple organisms providing protection against one or more antigens. MAPs are highly immunogenic thus inducing antibody responses capable of neutralizing receptors or invasion associated proteins in virus like dengue [83], bacteria like anthrax [84], or even tumors [85]. The plethora of information generated with regard to* A. marginale* outer membrane proteins so far tested in the laboratory and even those already used for animal inoculation [81, 86, 87], and other proteins yet to be tested, could be used for designing MAPs which can easily be assayed in the laboratory for immunogenicity against sera (IgG2) or even lymphocytes of from animals experimentally or naturally immunized.

## 6. New Omics Perspectives

More recently, genomic and proteomic approaches have facilitated the identification of minor components of the bacterial outer membrane that could be used as vaccines, so far the most effective mean to control infections in humans and animals [74, 78, 88]. Thus, omics approaches represent an alternative that may give information about* A. marginale* so far unknown.

New methodologies such as metabolomics profiling are, for example, a helpful tool to identify those metabolites involved in the induction of immune protection after vaccination which may have a potential use as a candidate vaccine and provide novel perspectives to vaccine design. Gray et al. [89] identified metabolites in the plasma of calves vaccinated with an intranasally delivered respiratory vaccine. Furthermore, metabolites may play important roles as ultimate end stage products or mediators of biological processes, so the analysis of the metabolites present in bovine plasma is the result of the mucosal immune response of the host.

While new information is continuously generated to identify possible vaccine candidates against bovine anaplasmosis, today there are no commercial vaccines in the world, and the efforts must continue to gain a better understanding of* A. marginale* and its relationship with their hosts (mammalian and tick) in order to have a vaccination program in the near future.

Development of new vaccines and therapeutics has been driven mainly by the understanding of the pathogenesis of infectious agents; however, the development of vaccines of many pathogens, including* A. marginale*, remains elusive [90]. Today, it is clear that the antigens used do not necessarily have to be virulence factors and other proteins have been identified by “omics” techniques. For instance, transcriptomics and proteomics analyses enable the identification of array of antigens expressed by a pathogen under specified conditions, by examining mRNA and proteins, respectively. When this analysis is made on the subset of proteins located in the surface of the pathogen, we refer to this as surface proteome; also, we can analyzed genes that are functionally important for infection by functional genomics [91].

The surface proteome represents information particularly important in order to understand the induction of protective immunity in the mammalian host and the transition from the mammalian host to the tick vector. A proteomics analysis using liquid chromatography and tandem mass spectroscopy (LC-MS/MS) revealed the idea that the surface complexes of* A. marginale* isolated from erythrocytes of the mammalian host were composed of multiple membrane proteins, most of which belong to protein family pfam 01617, which is conserved among members of the closely related genera* Anaplasma* and* Ehrlichia* [76]. On the contrary, the surface proteome of* A. marginale* isolated from tick cells was less complex and contained a novel protein, AM778, not identified in the surface proteome obtained from erythrocytes of the mammalian host [76].

Studies in* A. marginale* isolated from erythrocytes showed a number of proteins identified including Omp1, Omp7–9, Omp11, Msp1a, Msp2–4, OpAG2, Am1011, Am780, Am779, Am854, VirB10, while in* A. marginale* isolated from tick cells some of the proteins were Msp2–4 and Am778 [76]. These results support the contention that different proteins are expressed in* A. marginale* surface when it is in the mammalian host or in the tick, which open possibilities to new targets.

Today, almost any cellular condition can be analyzed, and new fields of study have arisen. Through immunomics, we can elucidate the set of antigens that interact with the host immune system and the mechanisms involved in these interactions; structural vaccinology reveals the structural epitopes of immunogenic antigens and vaccinomics explains the way in which the host's immune system responds to vaccines [91, 92].

Vaccinomics is based on the use of the genome-scale or “omics” technologies and bioinformatics for the development of next generation vaccines and refers to the “integration of immunogenetics and immunogenomics with systems biology and immune profiling” [93–95]. Vaccinomics is a holistic field that can take advantage of the information derived from the immune responses network theory and applies this information to the practical aspects of conceiving, designing, and delivering new vaccine candidates, which in turn is based on a better understanding of the key drivers of the immune system response to antigens at systems level in the host as well as advances in the understanding of genetic and nongenetic drivers of the immune response [96].

The main goals of vaccinomics or systems vaccinology [97] are, on one hand, the development of new vaccines through understanding the global architecture of the host immune response and the changes that occur following vaccination and, on the other hand, defining the signatures of protection required to elicit a protective immune response [94].

de la Fuente and Merino [98] have proposed the use of vaccinomics methodology as an alternative for developing new tick vaccines. First, they proposed the characterization of tick-host-pathogen interactions through genomics, transcriptomics, proteomics, metabolomics, and immunogenomics in order to perform data integration and a further analysis. Then, the development of algorithms that allow the identification of protective antigens in this plethora of information is critical for the formulation of candidate vaccines and its validation.

This strategy could be time-consuming and expensive and requires bioinformatics skills and trained personnel; however, the possibilities for the identification of candidate protective antigens and fulfilling the whole process of characterization and validation are a risk worth taking.

Vaccinology in the genome era has a wide repertoire of alternatives to analyze genomes. Out of these alternatives, pangenomic analysis compares the genomes of multiple isolates of a pathogen and those of close pathogenic and nonpathogenic relatives and bacteria of special interest. This analysis is not restricted to genome size, gene content, and gene conservation or variability among different strains, but it is also for the implications for effective vaccine and drug-discovery programs [91]. The pangenome concept is defined as the entire genomic repertoire of a given species or phylogenetic clade when multiple species are defined by systematics. The information provided by pangenomic analyses is divided into three groups: the core genes (shared by all genomes), the dispensable genes, and the strains (or isolate) specific genes [99–101].

Through pangenomic analyses, Dark et al. [72] found that* A. marginale* has a closed-core genome with few highly plastic regions including* msp2* and* msp3* genes and the* aaap* locus that appears to be expanding and contracting within and between strains. Although* A. marginale* genome sequence is highly conserved in gene content, it is also highly recombinogenic, which leads to plasticity. An example of this is* msp2* gene, which encodes a highly antigenic protein that varies over time during infection by gene conversion of functional pseudogenes into a single expression site to create new antigenic variants capable of evading host immune response. Comparison of St. Maries genome with Florida strain genome showed that Florida's genome contains one additional* msp2* functional pseudogene and, out of the eight Florida* msp2* functional pseudogenes, four are identical to St. Maries'. In contrast, only two of the seven* msp3* functional pseudogenes are shared between Florida and St. Maries. Finally, no new genes were detected in the pyrosequenced contigs of any of the strains. Dark et al. [72] also compared five* A. marginale* strains (Florida, St. Maries, Puerto Rico, Mississippi, and Virginia) that have differing abilities to be transmitted by* Dermacentor andersoni* ticks, with each phenotype represented by at least two geographically distinct isolations. The authors found that the number of single nucleotide polymorphism (SNP) between Puerto Rico, Virginia, and Mississippi strains is minimal (2,729, 3,868, and 6773, resp.); on the contrary, there were 9,609 SNPs between Florida and St. Maries strains, comprising 0.80% of the larger Florida genome. This indicates that the interstrain SNP diversity neither appears to be influenced by the environmental niche an organism occupies, nor is it generally consistent throughout a specific family or genera.

Analysis of multiple genomes provides a plethora of information that could help to better understand the organisms' environment and how adaptation exerts an important role in bacteria and its genome. We must emphasize the fact that as long as there exist* A. marginale* with different virulence phenotypes, pangenomic analyses are indispensable.

## 7. Conclusions

To this day, there are no commercial alternatives for the immunoprophylactic control of bovine anaplasmosis. Conventional vaccinology approaches have resulted in effective live attenuated or avirulent vaccines, which may also transmit other blood-borne pathogens or due to the inclusion of* A. centrale* may not be used in countries where the organism is absent.

Many molecular and bioinformatics-based studies have defined a number of surface membrane proteins and components of the type IV secretion system as potential antigens.

While the complete genome sequences of several* A. marginale* strains have been published, most of these sequences come from strains from the United States. Additional genomic information from strains from other countries is needed if a wide spectrum vaccine is to be designed and used over large regions of the world.

Many more genomic and other omics studier will be required in order to unravel the most relevant antigens to be used eventually, in a reliable vaccine for the control of anaplasmosis in large regions of the world.

## Figures and Tables

**Table 1:** MSP1a tandem repeats reported worldwide. The wide genetic diversity observed is result, in most cases, of cattle movements, veterinary practices, and vector population dynamics.

*Anaplasma marginale* strain	Structure of *msp1a* tandem repeats									Ref.
*Argentina*										
Virasoro	Σ	B	Q	B	C					[40]
Salta	B	B	M							
Entre Ríos 1	F	M	M							
Entre Ríos 2	F	M	M							
Entre Ríos 3	F	M	M							
Entre Ríos 4	F	M	M							
Santa Fe 37	*α*	*β*	*β*	*β*	Γ					
Santa Fe 43	*α*	*β*	*β*	*β*	Γ					
Santa Fe 50	*α*	*β*	*β*	*β*	Γ					
Santa Fe 59	*α*	*β*	*β*	*β*	Γ					
Santa Fe 111	B	B	M							
Santa Fe 473	B	B	M							
Santa Fe 532	B	B	M							
Santa Fe 116	B	B	M							
Chaco 2 var1	*τ*	22	13	18						
Chaco 2 var2	*α*	*β*	Γ	Γ	*β*	*β*	Γ			
Chaco 3 var1	*τ*	22	13	18						
Chaco 3 var2	*τ*	11	10	10	11	10	15			
Chaco 5	*τ*	10	15							
Chaco 7	*τ*	22	13	18						
Chaco 8	*τ*	22	13	18						
Córdoba 1	23	24	25	26	27	27				
Córdoba 2	23	24	25	26	27	27				
Quitilipi	28	29	m	29	M	F				
Mercedes	23	30	31	31	31					
Corrientes	*α*	*β*	*β*	*β*						

*Australia*										
Australia F12	8									[30]
Australia F72	8									
Australia Darwin	8									
Australia WA	8									

*Brazil*										
Minas-1	*τ*	57	*β*	*β*	*γ*					[33]
Minas-2	Is9	24	24	25	31					
Minas-3	*α*	*β*	*β*	*γ*						
Minas-4	B	Q	B	M						
Minas-5	13	27	27	27						
Minas-6–10	72	62	61							
Minas-11	*τ*	57	13	18						
Minas-12	72	62	61							
Minas-13	*α*	*β*	*β*	13						
Brazil	B	B	Q	*μ*						
Brazil 5	C	F	N							
Brazil 9	*α*	*β*	*τ*	M						
Brazil 12	*α*	*β*	*β*	N						
UFMG-1	13	42	13	18						
UFMG-2	13	27	27							
Paraná	*α*	*β*	*β*	*β*	*β*	Γ				[43]
Paraná 2	16	F	17	13	18					
Paraná 3	*τ*	10	15							
Lins SP/7	*τ*	10	15							[35]
Lins SP/10	*τ*	10	15							
Lins SP/16	*τ*	10	15							
Lins SP/11	191	13	18							
Lins SP/12	*α*	*β*	*β*							
Lins SP/110	*α*	*β*	*β*	*β*	13					
Lins SP/703	*α*	*β*	*β*	192						
Lins SP/1136	*τ*	*β*	100							
Lins SP/1228	*α*	*β*	*β*	Γ						
Lins SP/1450	193	*β*	100							
Lins SP/1453	191	13	Γ							
Mambaí GO/1017B	*α*	*β*	*β*	*β*	Γ					
Mambaí GO/1568B	*α*	*β*	*β*	*β*	Γ					
Mambaí GO/1806B	E	F	*φ*	*φ*	F	F				
AmRio1	162	F	17	F	F					[36]
AmRio2	*α*	*β*	*β*	*β*	F					

*Canada*										
Canadian bison	D	Q	Q	R						[30]

*China*										
HB-A8	19	20	19	21						[30]

*Cuba*										
Havana	A	B	B	B	B					[34]

*Israel*										
Israel tailed 1FM3	1	F	M	3						[30]
Israel nontailed	1	4								
Israel tailed 12M3	1	2	M	3						
Azaria	1	F	M	3	3					
Lhavot-Habasan	M	F	F	F						
Or-Haner	M	F	F							

*Italy*										
Italy 6	Q	M	Q	Q	M					[30]
Italy 8	Q	N	N	N						
Italy 30	M	M	M	Q						
Italy 31	M	M	M	Q						
Italy 32	5	Γ	Γ	Γ						
Italy 47	6	7	7	7						

*Mexico*										
Mex-31-096-01	T	C	B	B	C	B	*π*			[32]
Mex-30-130-01	T	C	B	B	C	B	C			
Mex-30-184-03	T	C	B	B	C	C				
Mex-15-099-01	*α*	*β*	*β*	Γ						
Mex-17-030-01	*α*	*β*	*β*	Γ						
Mex-30-193-01	*α*	*β*	*β*	Γ						
Mex-18-017-01	*α*	*β*	*β*	Γ						
Mex-07-068-01	*α*	*β*	*β*	Γ	*β*	Γ				
Mex-07-068-02	*α*	*β*	*β*	Γ	*β*					
Tamaulipas 17, 18	*α*	*β*	*β*	*β*	Γ					[41]
Mex-07-065-01	*β*	*β*	*β*	*β*	Γ					[32]
Mex-30-184-02	73	*β*	*β*	*β*	Γ					
Tamaulipas 15 (G9)	*τ*	57	13	18						[41]
Mex-14-010-01	*τ*	57	13	18						[32]
Mex-28-037-01	*τ*	57	13	18						
Mex-28-037-02	28	29	74	29	M	F				
Mex-30-184-01	72	C	F							
Mex-31-089-01	F	M	M							
Mex-17-017-01	12	13	14							
Mex-01-001-01	4	9	10	11	9					
Tamaulipas 1 (G1)	56	57	58	59						[41]
Tamaulipas 13 (G2)	4	9	10	10	9					
Tamaulipas 4 (G3)	60	61	61	62	61					
Tamaulipas 7, 9, 10, 12 (G4)	4	63	63	27	12					
Tamaulipas 11 (G5)	67	68	63	27	12					
Tamaulipas 14 (G6)	69	61	70	71	61					
Tamaulipas 5 (G7)	64	65	D	65	66					
Tamaulipas 6, 8 (G8)	D	65	D	65	66					

*Philippines*										
Batangas	Ph1	*β*	*β*	Γ	*β*	*β*	Γ			[42]
	Me1	4	M	M	4	4	4			
	Ph11	Ph11	Ph11	Ph11	M					
	Ph1	27	27							
	13	13								
	13	27								
	46	F								

Cebu	13	13	14	14	13	14	14			
	Ph4	17	Ph5	Ph6	Ph5	Ph7				
	13	13	13	14	14					
	Ph12	M	Ph12	M	M					
	13	13	13	MGl10						
	Ph9	Is1	Is1	Ph10						
	13	14	14							
	13	27	14							
	13	27	27							
	21	M	M							
	46	Ph20	46							
	13	27								
	13	MGl10								
	46	46								
	46	F								
	14									
	17									
	Me1									
	Ph8									
	13									

Iloilo	Ph4	17	Ph5	Ph7	Ph5	Ph7				
	Ph12	M	3	3	M					
	Ph4	17	Ph5	Ph5	Ph7					
	Me1	4	4	4						
	Ph16	Ph17	MGl10							
	Ph19	M	F							
	13	27	13	14						

Negros Occidental	Me1	4	M	M	4	4				
	Ph21	62	61	62	61	62				
	Me1	4	M	M	4					
	Ph2	Is1	Is1	Is1						
	Ph18	MGl10								
	Ph3									
	13	14								

Negros Oriental	Ph4	17	Ph5	Ph7	Ph5	Ph7				
	13	27								

*Puerto Rico*										
Puerto Rico	E	Φ	Φ	Φ	Φ	Φ				[30]

*South Africa*										
SA12	34	13	4	37						[30]
SW82	34	13	4	37						
SW62	34	13	4	37						
SW162	34	13	4	37						
SW134	34	13	4	37						
SA66	34	13	4	37						
SA193	34	4	37							
SW32	34	13	13	37						
SA14	34	F	4	H						
SA10	33	35	35							
SW29	3	3	38							
SA71	3	3	38							
SA302	3	3	38							
SA196	3	3	38							
SW114	3	13	4	4	37					
SW109	27	4	13	13	37					
SW44	27	4	4	4	37					
SW90	27	13	4	13	4					
SA239	27	4	13	4	4					
SA183	27	13	4	44						
SW34	34	45	45	46	37					
SA191	27	37								
SA189	27	37								
SA4	27	18								
SA63	39	37	13	13	13	13	37			
SA240	40	Q	Q							
SW113	41	13	13	13	4	37				
SW112	42	43	25	31						
SA243	3	36	3	36	36	3	36	38		
LP-7	34	159								[37]
LP-10	27	13	3	36						
LP-30	27	13	3							
LP-34	34	13	3	38						
LP-37	27	13	13	37						
LP-46	3	38								
LP-50	34	13	13							
MP-C2	34	13	158	37						
MP-C5	15	15	100	83						
NW-C2	27	13	4	4	37					
NW-C4	27	13	4	37						
NW-C5	82	13	79	4	37					
NW-CA-160312	34	13	3	36	38					
NW-C4-160312	34	36	38	3						
GP-C1	82	13	4	4	37					
GP-C2	34	27	3	38	13	3	38			
GP-C5	3	4	4	4	37					
GP-C112105	34	37								
GP-C4117105	3	36	38							
GP-C7117105	34	13	13							
GP-C1817105	34	13	37							
KZN-D	42	43	25	161	31					
KZN-F	42	43	25	31	31					
KZN-K	27	13	4	4	37					
KZN-Y	143	144	145	146						
KZN-MM	42	43	25	31						
KZN-14	142	43	25	31						
KZN-19	141	140	140							
KZN-49	147	148	149	150						
KZN-51	147									
EC-22	27	13	4	4	37					
EC-23	151	152	4	4	153					
EC-24	27	13	4							
WC-4	40	Q	Q	m						
WC-6	3	4	4	37						
WC-7	M	M	M	M						
WC-8	34	4	37							
WC-10	154									
WC-11	40	Q	Q	Q	Q	37				
WC-12	27	13	37							
WC-12	M	Q	M	Q	M					
WC-14	155	36	38							
WC-15	160	13	37	4	161					
WC-16	34	13	4	13	13	4	37			

*Spain*										
Va-48	40	47	47	32	C	C				[30]

*United States*										
Florida	A	B	B	B	B	B	B	B		[30]
California	B	B	C							
Okeechobee, FL	L	B	C	B	C					
Illinois	M	N	B	M	H					
Idaho	D	D	D	D	D	E				
Virginia	A	B								
Wetumka, OK	K	C	H							
Cushing, OK	L	C	B	C						
Cushing 2, OK	K	N	N	F	H					
Glencoe 1, OK	K	F	N	F	H					
Glencoe 2, OK	B	M	F	H						
Glencoe 3, OK	T	B	C							
Stillwater, OK	K	F	F	F	H					
Stillwater 2, OK	L	B	C	C						
Stillwater 68, OK	K	B	M	F	H					
Stillwater 483, OK	K	B	M	H						
Oklahoma City, OK	U									
Okmulgee, OK	K	B	V	C						
Stigler, OK	T	B	B	C						
Pawhuska, OK	I	H								
New Castle, OK	L	B	C	B						
St. Maries	J	B	B							
Mississippi	D	D	D	D	E					
Oregon	G									
Oregon, Rasmusen	A	F	H							
US bison (buffalo)	K	B	M	F	W					
Washington	B	B	B	C						
Missouri	B	B	B	B						
Texas	O	B	M	P						
Texas 198	B	B	m	B	m					
South Dakota	A	F	H							
Kansas 3261	B	B								
Kansas 4102	B	B	B							
Kansas 2267	B	B	B	B						
Kansas 0141	B	B	B	B	B					
Kansas 0063	B	B	B	B	B	B				
Kansas 5076	D	D	D	D	D					
Kansas 7042	D	D	E							
Kansas 4318	D	D	D	D	D	E				
Kansas 2070	D	D	D	D	D	D	E			
Kansas 7030	D	D	D	D	D	D	D	D	D	
Kansas 0050	E	M	Φ							
